# Tin-Doped Inorganic Amorphous Films for Use as Transparent Monolithic Phosphors

**DOI:** 10.1038/srep11224

**Published:** 2015-06-10

**Authors:** Hirokazu Masai, Hiroki Miyata, Yasuhiro Yamada, Shun Okumura, Takayuki Yanagida, Yoshihiko Kanemitsu

**Affiliations:** 1 Institute for Chemical Research, Kyoto University, Gokasho, Uji, Kyoto 611-0011, Japan; 2 Kyushu Institute of Technology, 2-4 Hibikino, Wakamatsu-ku, Kitakyushu 808-0196, Japan

## Abstract

Although inorganic crystalline phosphors can exhibit high quantum efficiency, their use in phosphor films has been limited by a reliance on organic binders that have poor durability when exposed to high-power and/or high excitation energy light sources. To address this problem, Sn^**2+**^ -doped transparent phosphate films measuring several micrometers in thickness have been successfully prepared through heat treatment and a subsequent single dip-coating process. The resulting monolithic inorganic amorphous film exhibited an internal quantum efficiency of over 60% and can potentially utilize transmitted light. Analysis of the film’s emissivity revealed that its color can be tuned by changing the amount of Mn and Sn added to influence the energy transfer from Sn^**2+**^ to Mn^**2+**^. It is therefore concluded that amorphous films containing such emission centers can provide a novel and viable alternative to conventional amorphous films containing crystalline phosphors in light-emitting devices.

Recent advances in light-emitting diodes (LEDs) have been accompanied by the development of new phosphors^1^,^2^,^3^,^4^,^5^ for light-emitting devices^6^,^7^,^8^,^9^,^10^,^11^ the luminescence of which depends on the emission center used and its surrounding matrix. At present, virtually all practical light-induced emission devices are predominantly fabricated from a mixture of powdered phosphors and organic binders^12^,^13^,^14^; however, the damage caused to organic binders such as silicone resins by high-power and/or high-energy excitation light sources (*e.g.*, high power LEDs) tends to reduce their service life. Furthermore, light scattering at the interface between the powdered phosphors and the surrounding matrix is not inherently eliminated in conventional devices that consist of a crystalline phosphor and binder. Consequently, transparent inorganic films capable of light-wave conversion will likely be required for white LED or sunlight converter applications in the near future. In particular, monolithic inorganic (and ideally amorphous) materials possessing good emissivity will be required for industrial applications of large-area devices.

A solution to this lies in the preparation of monolithic, inorganic bulk-glass phosphors containing an emission center. Since such a structure has no grain boundaries between the emission center and surrounding glass, the loss of external efficiency is expected to be greatly reduced. Investigation into the emission properties of amorphous glasses has so far been mainly limited to rare earth (RE)-containing glasses^15^,^16^,^17^,^18^,^19^ whose emission is almost independent of the coordination state compared to other emission centers; however, uncertainty as to the future stability of RE supplies has seen RE-free materials extensively examined in other fields. Thus, it would appear that a monolithic RE–free material possessing good emission properties is what will be required for future applications.

If we consider the transition probability of emission centers then a parity-allowed transition is favorable to attaining high emission intensity. For instance, a non-RE *ns*^2^-type emission center (*n* ≧ 4) possessing ns^2^-nsnp transition^20^,^21^,^22^,^23^,^24^,^25^,^26^,^27^ has a sufficiently high transition probability for use a practical phosphor, as is seen with Sb^3+^ - or Mn^2+^ -doped calcium halophosphate^27^. Our group has therefore focused on *ns*^2^-type cations, and has previously reported blue light emission from Sn^2+^ -doped ZnO-P_2_O_5_ glass^28^,^29^. Various light emissions have also been reported from a MnO-doped SnO-ZnO-P_2_O_5_ glass^30^,^31^, including an intense UV-excited emission comparable to that of a crystal phosphor such as MgWO_4_. Of particular note was the finding that a transparent RE-free bulk oxide glass attains high quantum efficiency, suggesting that despite being typically thought of as *passive* materials with regards to light, they may in fact have great potential for *active* light conversion^28^,^29^,^30^,^31^,^32^. Although the use of bulk glass presents one solution to providing a suitable light conversion technique, it is poorly suited to applications in which it is desirable to utilize established techniques for the fabrication of light-emitting devices such as white LEDs or solar cells. Consequently, there is a need to develop methods for preparing thin-film phosphors that can be readily adapted to large-area devices.

Broad emission from a Sn^2+^ center in a SnO-ZnO-P_2_O_5_ amorphous thin film under excitation with UV light has been recently reported^33^ with this film being produced from a precursor solution of SnCl_2_, ZnCl_2_, 85% H_3_PO_4_, and ethanol by dip-coating and heat-treatment. When compared with conventional sol-gel techniques, this method yields a much greater film density and a thickness of several microns; however, both the flatness and transparency of the film were insufficient due to the evaporation of the solvent or water. Thus, in order to improve the film condition, we have since focused on a solvent-free acid–base reaction that was previously used for the preparation of organic-inorganic hybrids^34^. In this technique, a spontaneous reaction between Si-Cl and P-OH forms a Si-O-P network through the elimination of HCl, which suppresses foaming and produces a remarkable volume shrinkage during heat treatment. It is expected that transparent inorganic amorphous films possessing photoluminescent (PL) properties can be obtained using this solvent-free technique, and in this study is used to prepare Sn^2+^ -containing oxide phosphor films. By studying the luminescence properties of these films, this report provides a valuable benchmark for transparent and highly photoluminescent inorganic films produced using an industrially viable fabrication technique.

## Results

### Preparation and examination of emission properties

**Figure 1:**
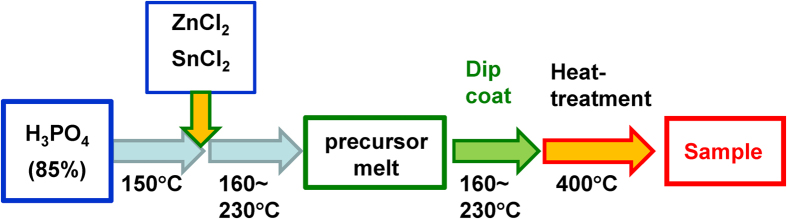
Preparation method for a SnO-ZnO-P_2_O_5_ film.

**Figure 2:**
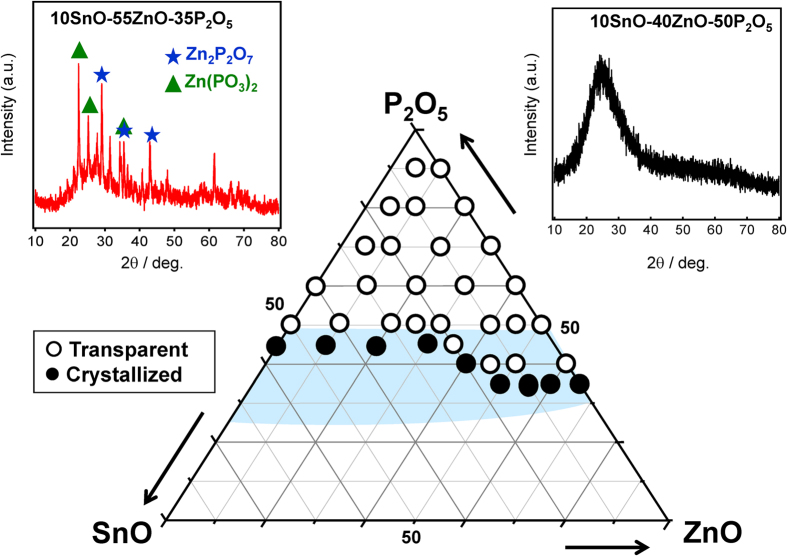
SnO-ZnO-P_2_O_5_ ternary amorphous film system, together with the bulk glass formation region reported by Morena^35^. The open and closed circles indicate amorphous and crystallized films, as shown in the typical XRD patterns.

**Figure 3:**
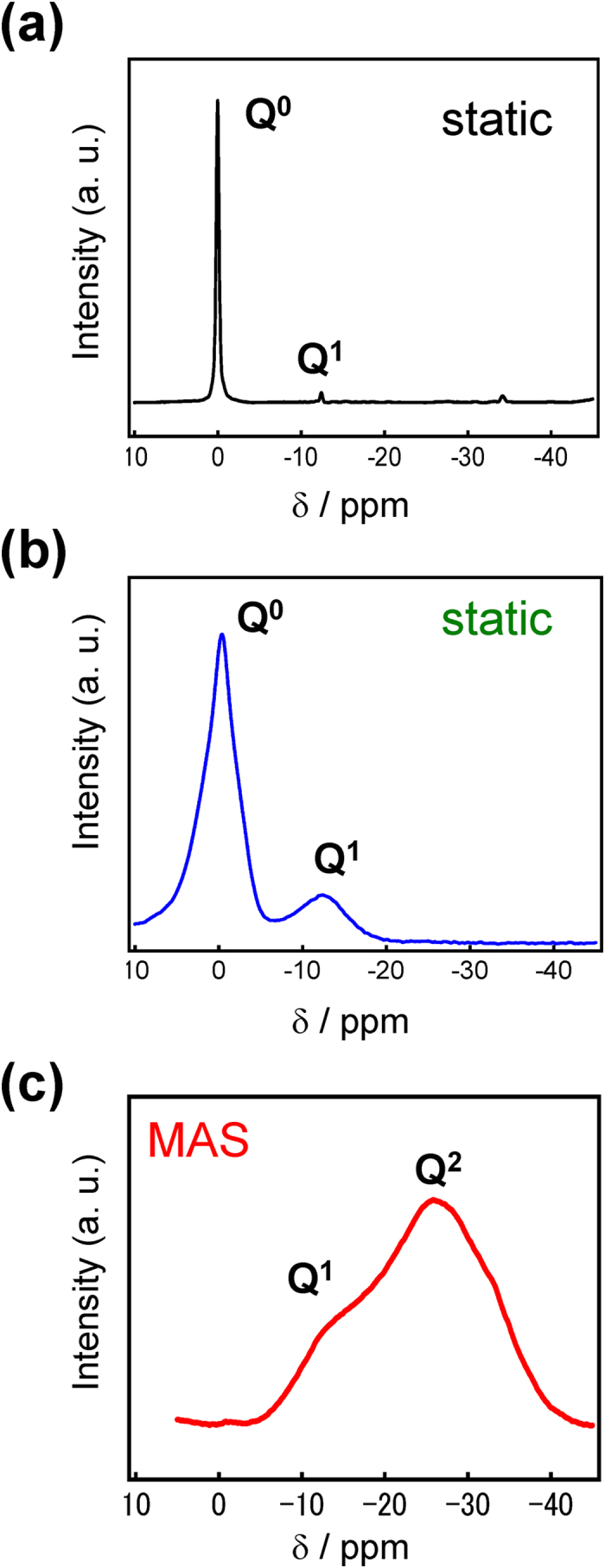
^31^P NMR spectra of a 10SnO-40ZnO-50P_2_O_5_ precursor melt and amorphous film after heat-treatment. (**a**) Precursor melt prior to addition of chlorides. (**b**) Precursor melt prior to dip coating. (**c**) Amorphous film.

The transparent film formation region of the SnO-ZnO-P_2_O_5_ ternary film system was first investigated according to the preparation scheme shown in Fig. 1, while the film system itself shown in Fig. 2 along with the bulk glass formation region reported by Morena^35^. This solvent-free preparation technique resulted in a composition that is at least 40 mol% P_2_O_5_, but the region in which this amorphous film is formed differs notably from that of bulk glass prepared using conventional melt-quenching methods^35^. Indeed, several samples were observed to turn opaque following heat treatment, which was attributed to the crystallization of zinc phosphate phases such as Zn_2_P_2_O_7_. Figure 3 shows the ^31^P Nuclear Magnetic Resonance (NMR) spectra of a 10SnO-40ZnO-50P_2_O_5_ precursor melt and an amorphous film produced from it by heat-treatment, the former consisting of Q^0^ units (Fig. 3(a)) prior to chloride addition that are also clearly observed in the precursor melt prior to dip coating (Fig. 3(b)). The generation of phosphate chain Q^2^ units (Fig. 3(c)) during heat treatment is therefore confirmed; but since it is often reported that phosphate units result in low water durability, whichever chemical composition produces the least amount of P_2_O_5_ is clearly the most favorable. Thus, in order to examine the relationship between the chemical composition and PL properties, we selected the *x*SnO-(50-*x*)ZnO-50P_2_O_5_ (mol%) system and prepared transparent films with various SnO:ZnO molar ratios. Table 1 shows the chemical composition of SnO-ZnO-P_2_O_5_ films obtained by energy dispersive X-ray (EDX)-Scanning Electron Microscope (SEM) measurement, which confirmed that there is only a small difference between the nominal and actual composition.

**Figure 4:**
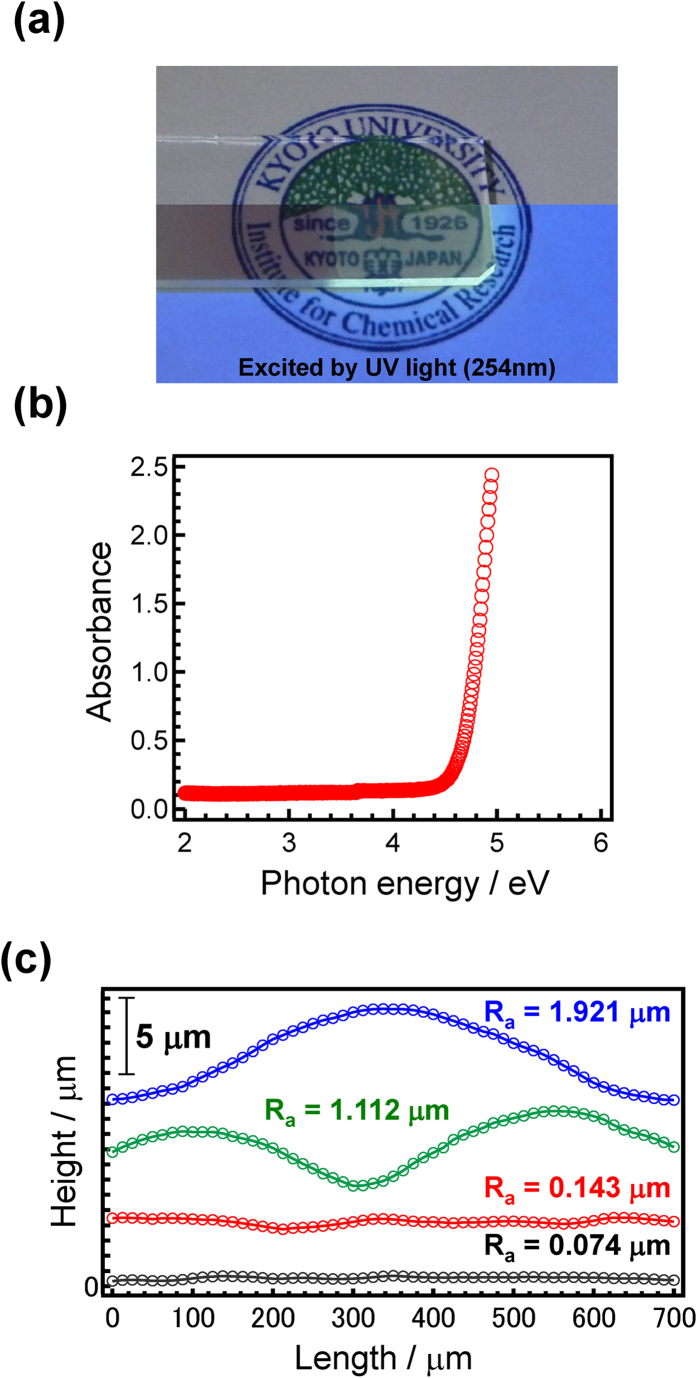
Optical properties of a SnO-ZnO-P_2_O_5_ amorphous film. (**a**) Photograph of a 10SnO-40ZnO-50P_2_O_5_ film with and without UV irradiation (4.88 eV). The clearly visible blue light emission is due to the Sn^2+^ center. (**b**) Optical absorption spectrum for a 10SnO-40ZnO-50P_2_O_5_ film 10 μm in thickness. (**c**) Surface profiles of the 10SnO-40ZnO-50P_2_O_5_ film at different measurement points.

Most of the amorphous films produced were found to be colorless and transparent both by visual observation (Fig. 4a) and from their optical absorption spectra (Fig. 4b, and supplemental figure 1). Since conventional deposition techniques for inorganic films such as dip- or spin-coating produce films with a submicron scale thickness, even with the addition of a thickener, such techniques are difficult to adapt to producing micron-thick films suitable for photon energy conversion. Moreover, conventional procedures inherently require coating or heat-treatment cycles to obtain a thick film, whereas we have confirmed that a slower elevation rate is all that is needed to achieve film thickness in the order of several micrometers. Indeed, the viscous nature of the precursor melt used in this study allows films several microns in thickness to be obtained by using just one dip-coating and heat-treatment procedure (*i.e.* a single dip-heat-treatment process). The TEM and SEM images are shown in supplemental fig. 2. From these images, it is found that the amorphous thick film without crystallites or cracks was successfully obtained. Figure 4c shows the surface profile of the 10SnO-40ZnO-50P_2_O_5_ film at different points, with the upper part of the film exhibiting a thickness of about 10 μm (elevation rate: 1 mm/s) and a relatively smooth surface (*R*_a_ ~ 0.1 μm). The roughness (*R*_a_ ~ 1 ~ 2 μm) was, however, also observed in the lower part of the film due mainly to the increase in film thickness during the dip-coating process. Furthermore, the transparent film exhibited a clear blue-light emission under irradiation by UV light, whereas no PL properties were observed in the case of a Sn-free sample. Thus, it is important to emphasize that the present coating method is quite different from existing techniques in that it can achieve micron-thick films with a single process that is suitable for industrial application.

**Figure 5:**
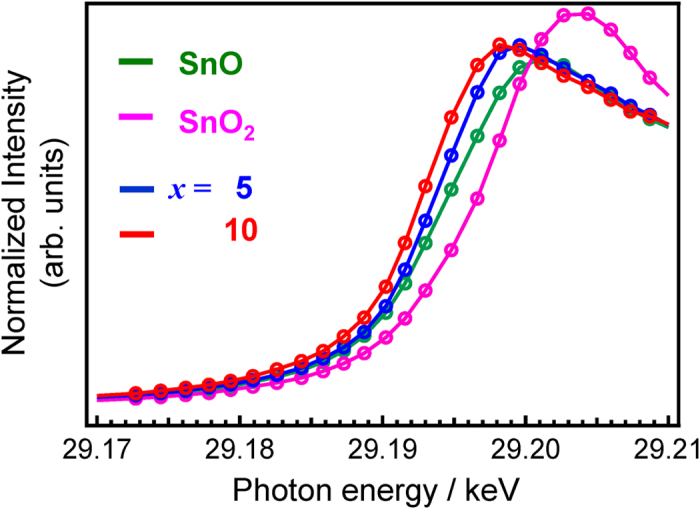
Sn K-edge XANES spectra of *x*SnO-(50-*x*)ZnO-50P_2_O_5_ films (*x* = 5 and 10). Also shown for comparison are the spectra for SnO and SnO_2_.

Because Sn^2+^ is a metastable species, and therefore easily affected by the preparation conditions^36^,^37^ it is important to examine the valence state of Sn in the matrix. Figure 5 shows Sn K-edge XANES spectra of several *x*SnO-(50-*x*)ZnO-50P_2_O_5_ films (*x* = 5 and 10), with the spectra for SnO and SnO_2_ also provided for reference. If we take the absorption edge energy, *E*_0_, to be the energy at the zero-intercept of the second derivative, we can evaluate the oxidation state of the Sn cation from the *E*_0_ value; |Δ(*E*_0_(SnO)-*E*_0_(film))| being calculated as less than 1.7 eV in all instances. Considering the resolution of the measurement (Δ*E*/*E* ~ 6 × 10^−5^: ~1.75 eV), it is assumed that the difference between values is insignificant. Thus, the Sn K-edge XANES spectra suggest that the percentage of Sn^2+^ to total Sn in *x*SnO-(50-*x*)ZnO-50P_2_O_5_ films is nearly 100%. This is consistent with results in the literature that suggest that a valence change of Sn^2+^ to Sn^4+^ occurs at temperatures over 650 °C in air^38^.

**Figure 6:**
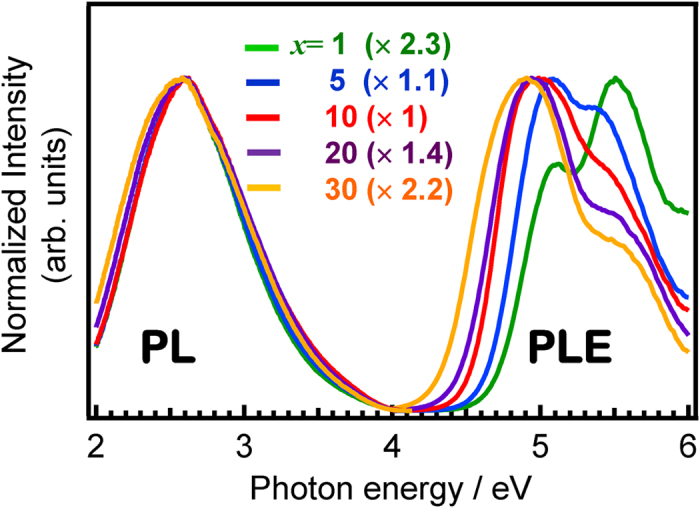
Normalized PL-PLE spectra of *x*SnO-(50-*x*)ZnO-50P_2_O_5_ films.

**Figure 7:**
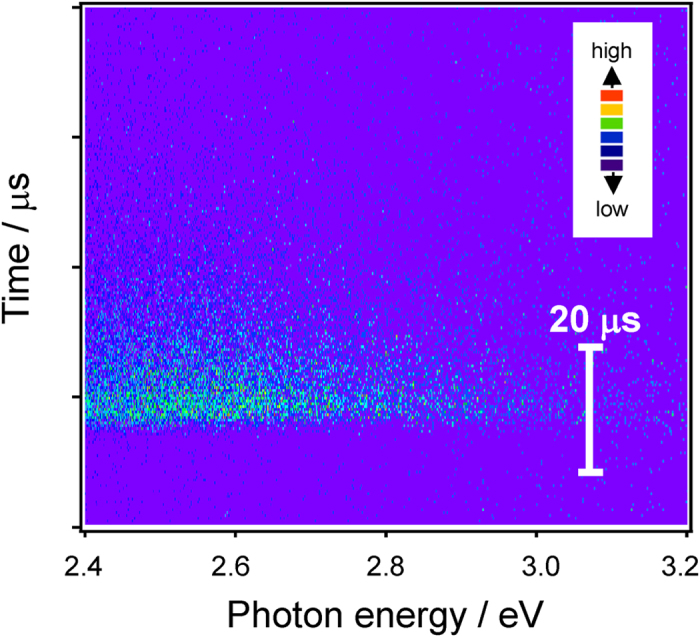
Streak image of a 10SnO-40ZnO-50P_2_O_5_ film obtained by excitation at 250 nm.

**Figure 8:**
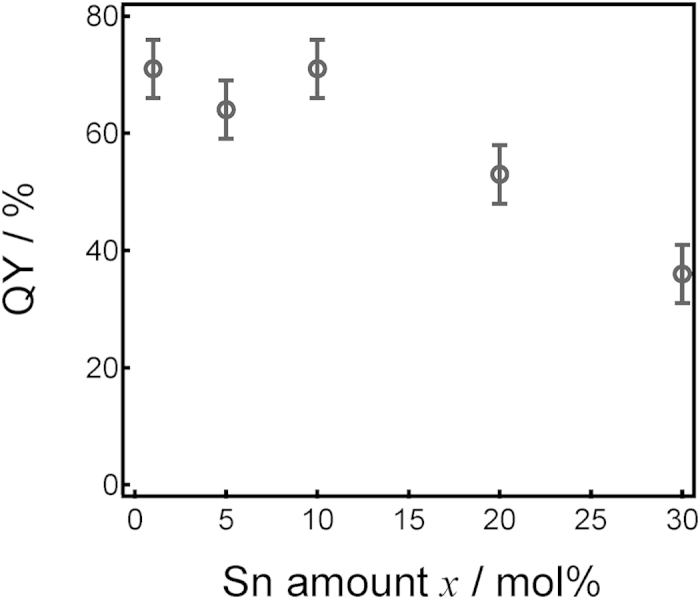
Correlation between internal quantum efficiency (QY) and the amount of SnO in *x*SnO-(50-*x*)ZnO-50P_2_O_5_ films.

Figure 6 shows PL-PLE spectra of *x*SnO-(50-*x*)ZnO-50P_2_O_5_ films with different SnO contents. In order to better understand the spectral change the intensities were normalized using the maximum value recorded, which was observed in the 10SnO-40ZnO-50P_2_O_5_ film. The PLE spectra were measured at the peak photon energy of each PL spectrum. The PL spectra show little dependence on the SnO concentration, but the excitation peak energy does slightly red-shift with increasing SnO concentration. Because the Sn^2+^ emission center possesses electrons in the outermost shell, its emission spectrum is strongly affected by the coordination field^20^,^36^,^39^. The unchanged emission spectra of Sn^2+^ therefore indicate that the local coordination states of the Sn^2+^ center are not so diverse, even though the host matrix is amorphous. Figure 7 shows a streak image of a 10SnO-40ZnO-50P_2_O_5_ film obtained by excitation at 250 nm, revealing that the timescale of film decay is in the order of microseconds and that it can be classified as a triplet–singlet relaxation of the Sn^2+^ center. The decay constant τ_1/e_ of the main band is about 8 μs, which is longer than that previously reported for bulk glasses^29^,^32^. Since this decay constant depends on the concentration, the observation that it remains the same suggests that the actual concentration in the Sn^2+^ center is below 1 mol%^29^. It is also notable that the emission peak energy does not shift to lower energies with increasing time, as was the case with Sn^2+^ -doped zinc oxide glass phosphors^29^,^32^. Although the local environment in glass materials can be estimated from the emission lifetime^40^,^41^, it is hard to determine some specific sites from the decay of the film. Therefore, we assume that Sn^2+^ emission centers exist with Gaussian distribution. On the basis of this emission spectra and streak image we believe that not all of the Sn^2+^ centers are good emitters/absorbers in the film, despite it containing 10 mol% SnO. In other words, only a limited number of the Sn^2+^ ions are capable of functioning as emission centers for PL. If the distribution of Sn^2+^ in bulk glass prepared by melt-quenching is also taken into consideration then the film can be expected to possess heterogeneity to at least a nanometer scale (phase-separated structure), which would explain why the concentration at which the fluorescence is quenched is around 10 mol% SnO. In other words, if we can control the local coordination state of the Sn^2+^ centers, then the chemical composition of the film can be drastically varied. Figure 8 shows the correlation between internal quantum yield (QY) and the amount of SnO in *x*SnO-(50-*x*)ZnO-50P_2_O_5_ films, revealing the QY of these films to be almost constant (over 60%) below 10 mol% SnO, but to decrease with increasing SnO content. This contrasts with previous reports on bulk glass phosphors^28^,^29^,^30^,^31^,^32^,^33^,^36^ in which more than a 20% decrease of QY is observed. As mentioned above, it is expected that some Sn^2+^ species do not function as an emission center, and so considering the relationship between the concentration of Sn^2+^ and the QY value, it is suggested that several Sn^2+^ species cluster to decrease the QY value. On the other hand, from the optical absorption spectrum, there is evidently sufficient excitation light absorbed by a film with a thickness of about 10 μm to ensure blue light emission.

### Demonstration of white light emission

**Figure 9:**
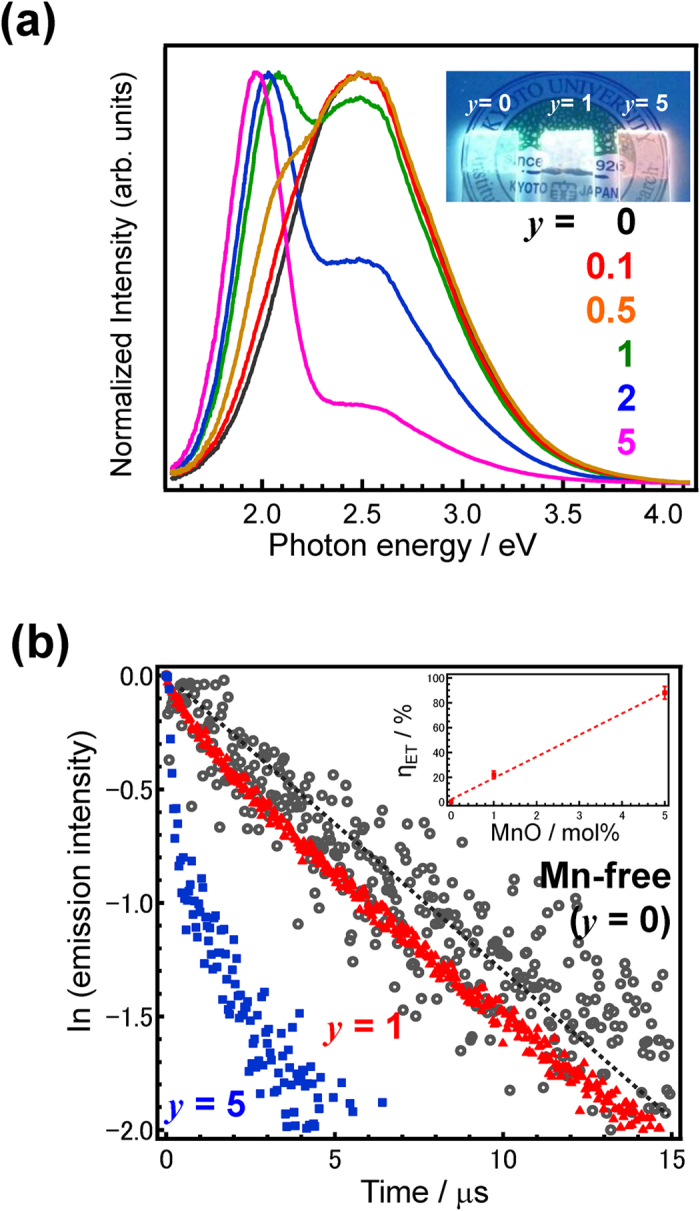
Emissive properties of a *y*MnO-10SnO-40ZnO-50P_2_O_5_ amorphous film. (**a**) Normalized PL spectra of a *y*MnO-10SnO-40ZnO-50P_2_O_5_ film excited at 4.96 eV. The inset shows a photograph of several films (*y* = 0, 1, and 5) under UV irradiation at 254 nm. (**b**) Emission decay curves of *y*MnO-10SnO-40ZnO-50P_2_O_5_ (*y* = 0, 1, and 5) films, as calculated from the integral of the photon number in the streak image within the 2.53–2.70 eV region. Inset shows the η_ET_ values as a function of MnO amount.

Figure 9a shows normalized PL spectra of *y*MnO-10SnO-40ZnO-50P_2_O_5_ films (where *y* = 0, 0.1, 0.5, 1, 2, and 5), while the inset shows photographs of several films (*y* = 0, 1, and 5) under 254 nm UV irradiation. These PLE spectra are shown in supplemental figure 3. Note that the color coordination positions continuously change from blue to red with the addition of MnO, and that these emission spectra consist of two broad bands attributable to the Sn^2+^ and Mn^2+^ centers that have also been observed in other materials^30^,^42^. Peak deconvolution using Gaussian function revealed that these two peaks show little change in their emission band or half-width, indicating that the local coordination of Sn^2+^ and Mn^2+^ is hardly affected by the MnO concentration. Moreover, the emission intensity of SnO is reduced when the amount of MnO is increased, suggesting that energy is transferred between them. In order to confirm this energy transfer emission decay curves of MnO-free and MnO-doped (*y* = 1.0, and 5.0) 10SnO-40ZnO-50P_2_O_5_ films were obtained, as shown in Fig. 9b, with the decay values being calculated from an integral of the photon number in the streak image within the 2.53–2.70 eV region. With the addition of MnO, the decay constant of the Sn^2+^ center with Mn^2+^ cations (τ_1/e_) is reduced, indicating that an energy transfer from Sn^2+^ to Mn^2+^ does occur. The energy transfer efficiency (*η*_ET_) is calculated from the τ_1/e_ values using the formula^43^:
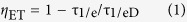
where τ_1/eD_ is the decay constant of a Sn^2+^ center without a Mn^2+^ cation. Using Equation (1), the *η*_ET_ values of the 1MnO-doped and 5MnO-doped 10SnO-40ZnO-50P_2_O_5_ films were found to be 21 and 88%, respectively (see inset of Fig. 9b). As these η_ET_ values are roughly dependent on the MnO concentration, the present data shows no evidence of the local Sn^2+^ -Mn^2+^ clustering structure that has been previously suggested to form in alkali halide^42^,^44^.

**Figure 10:**
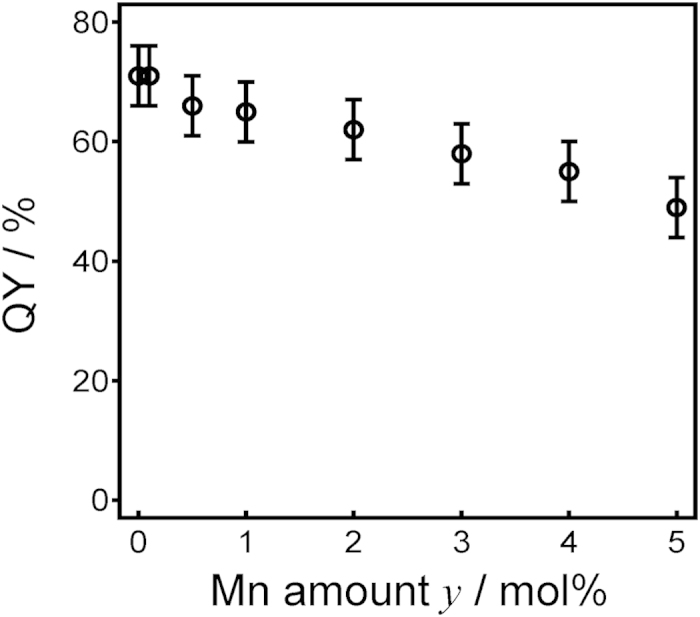
Correlation between QY and the amount of MnO in *y*MnO-10SnO-40ZnO-50P_2_O_5_ films.

Figure 10 shows the correlation between QY and the amount of MnO in yMnO-10SnO-40ZnO-50P_2_O_5_ films. The QY values of the MnO-doped films (*y* ≤ 2) were all over 60%; which, in combination with the similarity of their blue-white-red light emission to that of bulk glass, confirms their potential application as a transparent phosphor for large-area devices. Furthermore, it is possible to add other elements to these films to better control their emissivity (such as using metal halides like lanthanide-chloride to control the color) or to improve their mechanical and/or thermal properties. In particular, the recent development of a deep-UV LED^7^,^8^,^9^,^10^ suggests a very clear possibility for novel film-type emission devices in the near future.

## Discussion

In the present study, we have demonstrated the UV-excited light emission of Sn^2+^ -doped zinc phosphate films produced from a precursor melt by a solvent-free acid-base reaction. We have confirmed that the film can be also prepared on commercially available borosilicate or soda lime silicate (slide glass) plates. Importantly, this fabrication method allows inorganic films with a thickness on the order of several microns to be produced in a single dip-coating process. One of the main reasons for thick film is the high viscosity of the precursor melt containing no solvent. High-viscous precursor melt, whose viscosity depends on the temperature, make it possible to prepare thickness film without crack. This allows the transparent phosphor to utilise the energy of both converted and transmitted light. The presence of both phosphor crystals (micrometre size) and nanocrystallites also means that these monolithic films can reduce the optical loss of transmitted light produced by luminescence. Moreover, although their chemical durability is still insufficient for direct practical use owing to hygroscopicity, this could be potentially overcome by chemical or physical means such as a luminous layer in an organic LED (OLED).

The Sn^2+^ contained within the film exhibits an effective *T*_1_-*S*_0_ relaxation, this being despite the presence of nanoscale heterogeneity. Recently, we reported that the emission of the Sn^2+^ center does not reflect the average (macroscopic) basicity of the glass, but instead reflects the local basicity of the emission center^45^. This indicates that the macroscopic randomness and composition are not critical factors for attaining a high quantum efficiency, and that amorphous materials can exhibit high conversion efficiencies comparable to those of crystalline phosphors if the microscopic local coordination states of the emission centers are controlled. However, as pointed out earlier, not all Sn^2+^ species are a good emission center. Thus, although the local coordination state of a Sn^2+^ center needed to attain the highest light conversion has not yet been clarified, monolithic inorganic phosphor films exhibiting good emissivity can nevertheless be obtained by tailoring the local coordination state of the emission center. Therefore, the amorphous film exhibiting the internal quantum efficiency of over 60% can be improved from viewpoint of energy conversion. Moreover, its emission color can be fine-tuned by altering the energy transfer from Sn^2+^ to acceptors, such as Mn^2+^. These results confirm the validity of this approach, which is of great significance to the fabrication of transparent films exhibiting various light-wave shifts.

## Methods

### Preparation of SnO-doped zinc phosphate films

The starting materials for the SnO–ZnO–P_2_O_5_ film were SnCl_2_, ZnCl_2_, and 85% H_3_PO_4_. The film was prepared by conventional dip-coating at 160–230 °C using a precursor melt prepared by first heating 85% H_3_PO_4_ in a glass beaker to 120–150 °C for 3 h to remove any water or residual OH groups, after which SnCl_2_ and ZnCl_2_ were added at the same temperature. Next, the melt was gradually heated to 160–230 °C for 1 h under constant stirring in the ambient atmosphere to obtain a transparent homogeneous precursor melt. In the case of the Mn^2+^ co-doped samples, MnCl_2_ was added simultaneously with the SnCl_2_ and ZnCl_2_. In either case, a thin film of the melt was produced by dip-coating a pure SiO_2_ glass substrate at an elevation rate of 0.1 mm/s. Afterward, the sample was placed in an electric furnace under the ambient atmosphere at 400 °C for 10 min to obtain the final film.

### Analysis methods

The surface profile of the film was measured using a Surfcorder SE-30D (Kosaka Laboratory). Energy dispersive X-ray-SEM measurement was performed using a JSM-6500F microscope (JEOL). The ^31^P NMR spectra were measured using a CMX-400 NMR spectrometer (JEOL) at a frequency of 161.80 MHz, and the chemical shifts were estimated with respect to H_3_PO_4_ in a D_2_O solution (0 ppm). The ^31^P magic angle spinning (MAS) NMR spectrum was measured at a spin rate of 10 kHz and a pulse delay of 5 s, and a fine powder produced by pulverising the dip-coated glass was used for measurement in the case of the thin films. The PL and PLE spectra were measured at room temperature using an F-7000 fluorescence spectrophotometer (Hitachi) with appropriate band pass filters for the excitation (5 nm) and the emission (3 nm) required for PL measurements. The absorption spectra were measured from the centres of the films using a U3500 UV-vis-NIR spectrophotometer (Hitachi). The chemical composition of the films was determined by EDX-SEM measurement for a 500 nm spot size using a JSM-6500F (JEOL). Furthermore, XRD measurements were made to ascertain the crystallization behaviour of the film using a RINT2100 instrument (Rigaku). The emission decay at room temperature was evaluated using a streak camera in conjunction with a monochromator. The light source used for photoexcitation was an optical parametric amplifier system based on a regenerative amplified mode-locked Ti:sapphire laser (Spectra Physics) with a pulse duration of 150 fs and a repetition rate of 1 kHz. The photon energy was 4.96 eV, which corresponded to the peak energy of the PLE spectra. The Sn K-edge (29.3 keV) XAFS spectra were measured at BL01B1 of SPring-8 (Hyogo, Japan) using a Si (311) double crystal monochromator in the transmission mode (Quick Scan method) at room temperature. The storage ring was operated at an energy of 8 GeV with a typical current of 100 mA. The internal quantum efficiency was measured using a Quantaurus-QY (Hamamatsu Photonics), which possesses an integrating sphere in order to measure absolute efficiency. The errors of the measurement were ±3%.

## Additional Information

**How to cite this article**: Masai, H. *et al.* Tin-Doped Inorganic Amorphous Films for Use as Transparent Monolithic Phosphors. *Sci. Rep.* **5**, 11224; doi: 10.1038/srep11224 (2015).

## Supplementary Material

Supplementary Information

## Figures and Tables

**Table 1:** **Chemical composition of SnO-ZnO-P**_**2**_ **O**_**5**_ **films obtained by EDX-SEM measurement.**

**Nominal Chemical composition/mol%**	**Molar ratio measured by EDX SEM/mol%**		
	SnO (±1)	ZnO (±2)	P_2_O_5_ (±2)
5SnO-45ZnO-50P_2_O_5_	5	41	54
10SnO-40ZnO-50P_2_O_5_	8	38	54
15SnO-35ZnO-50P_2_O_5_	13	34	53
