# Supplementary material for: Tin-Doped Inorganic Amorphous Films for Use as Transparent Monolithic Phosphors
Source: Sci Rep. 2015 Jun 10;5:11224. doi: 10.1038/srep11224 (PMC4462154; doi:10.1038/srep11224)
Supplement: Supplementary Information [file srep11224-s1.pdf]

Tin-Doped Inorganic Amorphous Films for use as Transparent Monolithic Phosphors

H. Masai, H. Miyata, Y. Yamada, S. Okumura, T. Yanagida, and Y. Kanemitsu

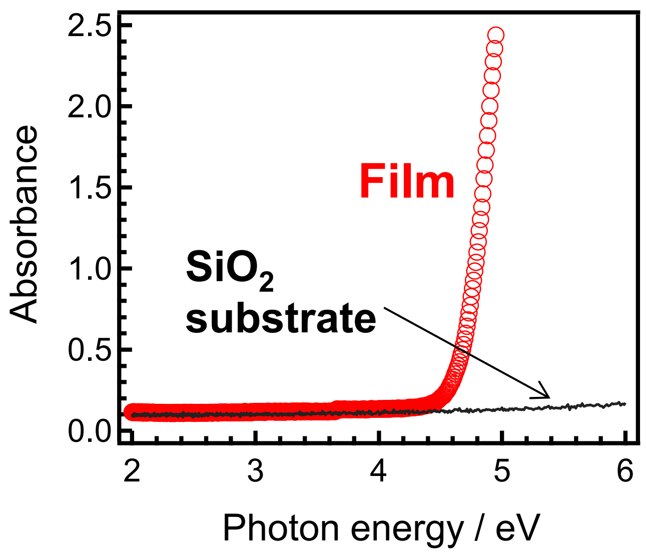

Supplemental Figure 1

Optical absorption spectra of a 10SnO-40ZnO-50P_2O_5 film and the SiO_2 substrate.

Tin-Doped Inorganic Amorphous Films for use as Transparent Monolithic Phosphors

H. Masai, H. Miyata, Y. Yamada, S. Okumura, T. Yanagida, and Y. Kanemitsu

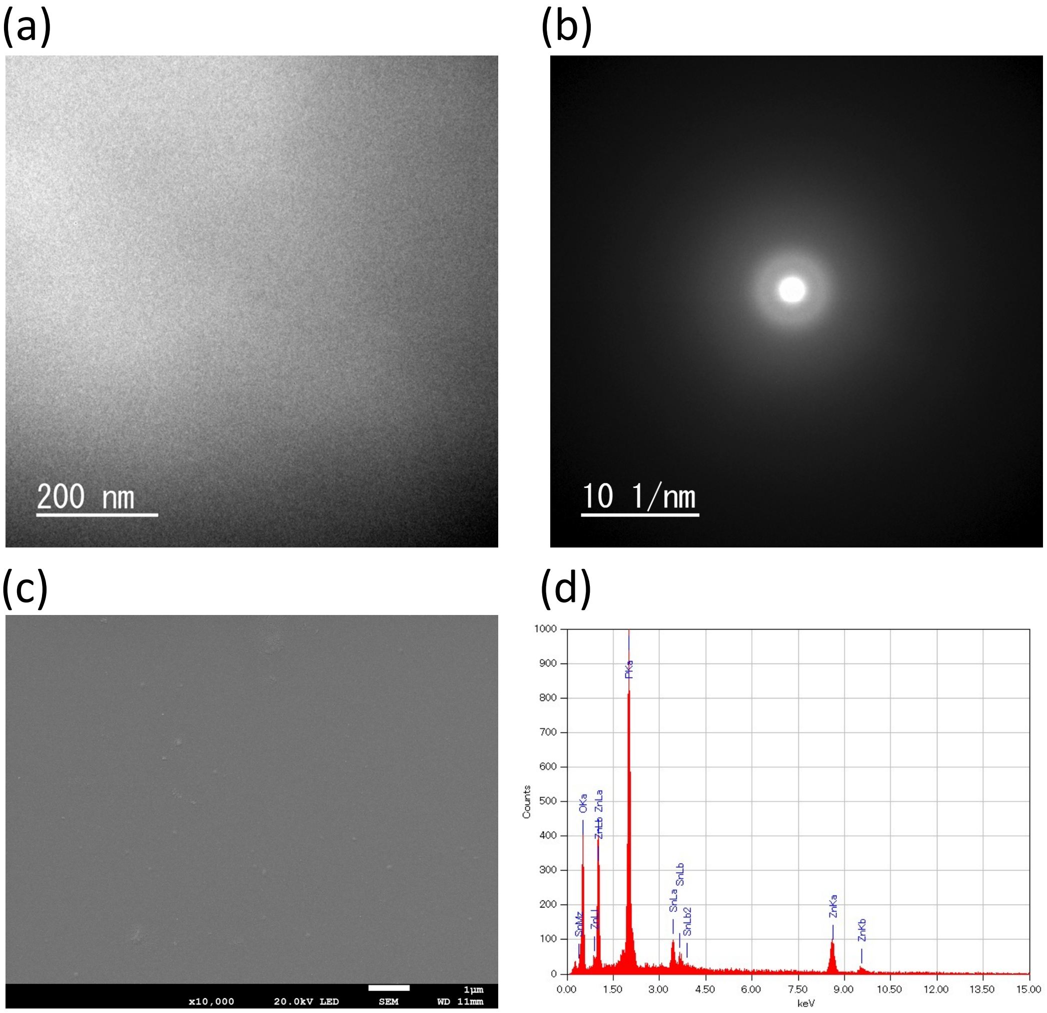

Supplemental Figure 2

TEM and SEM images of $10SnO$-$40ZnO$-$50P_2O_5$ film. (a) TEM image, (b) electron diffraction pattern, (c) SEM image, and (d) the EDX pattern of the film. Homogenous amorphous structure is observed.

Tin-Doped Inorganic Amorphous Films for use as Transparent Monolithic Phosphors

H. Masai, H. Miyata, Y. Yamada, S. Okumura, T. Yanagida, and Y. Kanemitsu

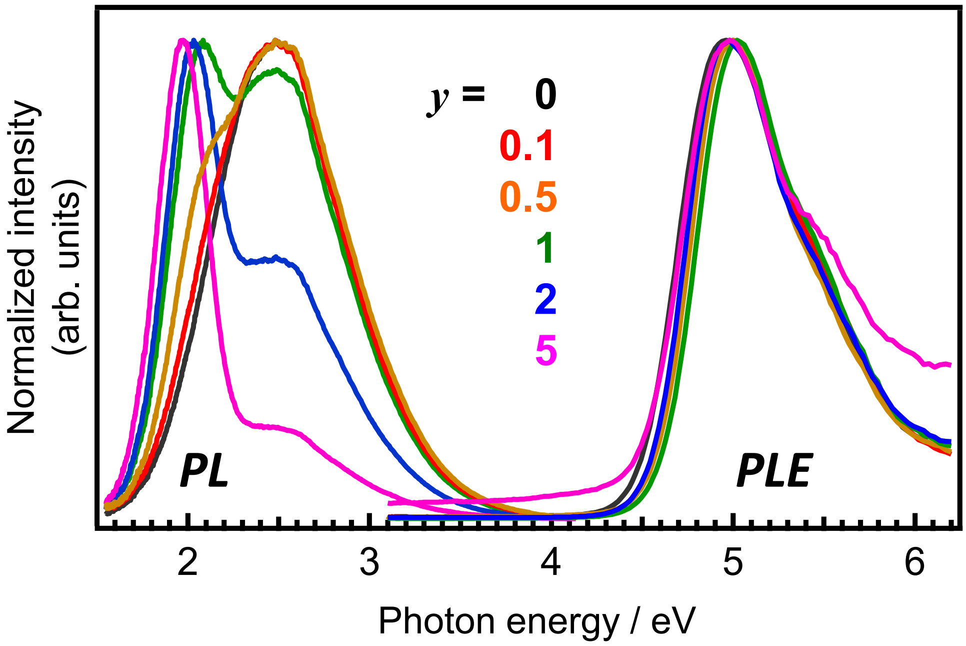

Supplemental Figure 3
PL and PLE spectra of *y*MnO-10SnO-40ZnO-50P_2O_5 films.
